# META-pipe cloud setup and execution

**DOI:** 10.12688/f1000research.13204.3

**Published:** 2019-05-02

**Authors:** Aleksandr Agafonov, Kimmo Mattila, Cuong Duong Tuan, Lars Tiede, Inge Alexander Raknes, Lars Ailo Bongo

**Affiliations:** 1 Department of Computer Science, UiT The Arctic University of Norway, Tromsø, Norway; 2 CSC - IT Center for Science, Espoo, 02150, Finland; 3 CESNET, Prague 6, 160 00 , Czech Republic; 4 Department of Information Technology, UiT The Arctic University of Norway, Tromsø, Norway; 5 Department of Chemistry, UiT The Arctic University of Norway, Tromsø, Norway

**Keywords:** ELIXIR, Portability, META-pipe, OpenStack, EGI Federated Cloud, Amazon Web Services, AAI federation, Apache Spark

## Abstract

META-pipe is a complete service for the analysis of marine metagenomic data. It provides assembly of high-throughput sequence data, functional annotation of predicted genes, and taxonomic profiling. The functional annotation is computationally demanding and is therefore currently run on a high-performance computing cluster in Norway. However, additional compute resources are necessary to open the service to all ELIXIR users. We describe our approach for setting up and executing the functional analysis of META-pipe on additional academic and commercial clouds. Our goal is to provide a powerful analysis service that is easy to use and to maintain. Our design therefore uses a distributed architecture where we combine central servers with multiple distributed backends that execute the computationally intensive jobs. We believe our experiences developing and operating META-pipe provides a useful model for others that plan to provide a portal based data analysis service in ELIXIR and other organizations with geographically distributed compute and storage resources.

## Introduction

ELIXIR was established to unite European life science resources. It has 21 member states and more than 180 research organizations that each take responsibility for an analysis service, database, software tool, training material, or provide cloud storage and compute resources. For example, one of the deliveries from ELIXIR Norway are marine metagenomics analysis services, whereas ELIXIR Finland provides cloud storage and compute resources. An ELIXIR user from Portugal may therefore use a service maintained in Norway run on resources in Finland. In this paper, we describe our approach for setting up distributed execution of such analyses.

META-pipe ^1^ is a complete workflow for the analysis of marine metagenomic data. It provides assembly of high-throughput sequence data, functional annotation of predicted genes, and taxonomic profiling. We provide META-pipe as an Analysis-as-a-Service for Norwegian and Finnish ELIXIR users. However, additional compute resources are necessary to open the service to all ELIXIR users. Users log into the META-pipe web application where they can upload data to analyze, select tool parameters, start analyses, and download analysis results. The functional annotation is computationally demanding and must therefore be run on a high-performance computing (HPC) cluster or a compute cloud. Job execution is handled by the META-pipe backend, such that resource allocation, parallel execution, and fault handling is hidden from the user.

**Figure 1.**
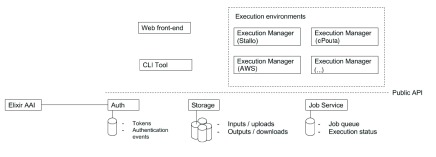
META-pipe backend architecture has three servers located at the University of Tromsø. The authorization server, which is integrated with the ELIXIR AAI, enables login for Elixir users. The storage server stores all META-pipe input, output and provenance data. The job server schedules and maintains submitted analysis jobs. The jobs are implemented as Spark programs that are executed by an execution manager running in an execution environment. There can be multiple execution managers distributed over many HPC clusters and clouds.

META-pipe has a distributed architecture with three central servers and geographically distributed execution managers ( Figure 1). We have currently four META-pipe execution managers: (i) the Sigma2 Stallo supercomputer in Tromsø, which is a Norwegian academic HPC; (ii) the CSC cPouta OpenStack based Infrastructure-as-a-Service cloud, Finland; (iii) the CESNET-MetaCloud OpenNebula cloud that supports the open cloud computing interface, Czech Republic; and (iv) the commercial Amazon EMR cloud service. cPouta is an ELIXIR compute service. CESNET-MetaCloud is part of the EGI Federated Cloud.

An important design goal for the META-pipe backend is to make the execution managers portable. In addition, we have taken care to make setup and maintenance of the execution managers easy. We achieve these goals since our backend is designed such that all state is maintained at the central servers. We therefore reduce the amount of code that needs to be ported, maintained, and optimized for a new execution environment. The execution managers are stateless, and the jobs are idempotent. This simplifies failure handling. We also use the widely available Apache Spark framework to execute the pipeline analyses. In addition, we have implemented tools that make it easy to set up and administer the execution managers. These pull the META-pipe tools, dependencies, and jobs from the centralized servers. In this paper, we describe these tools and their use. We make the following contributions:

1. We demonstrate the use of geographically distributed compute resources for life science data analysis.
2. We describe the design and implementation of cloud setup tools for our analysis service.
3. We describe our experiences developing and operating the META-pipe analysis service.

We designed our analysis service to be powerful, easy to use, and easy to maintain. We believe our work provides a useful model for others that plan to provide a portal-based data analysis service in ELIXIR and other organizations with geographically distributed compute and storage resources.

## Methods

**Figure 2.**
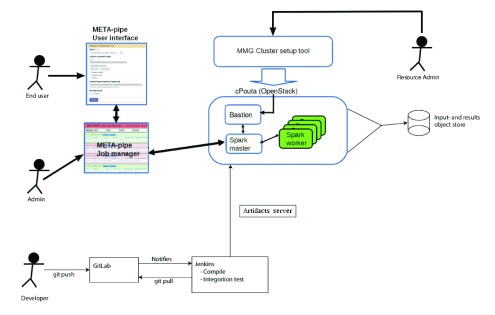
META-pipe deployment. End-users run analyses using the META-pipe web app. The web app is integrated with ELIXIR AAI, so users can authenticate using their home institution username and password. Resource providers use the cluster setup tool to set up an execution manager, on, for example, the cPouta OpenStack cloud, which executes analysis jobs. The execution manager, pipeline, and dependencies are all read from our artifacts server. META-pipe developers use git to maintain the code. Our GitLab is integrated with Jenkins that compiles and runs integration tests and pushes new META-pipe versions to the artifacts server. META-pipe administrators administer all jobs using the META-pipe Job manager interface.

The META-pipe is deployed as shown in Figure 2. META-pipe is implemented as a Spark program and requires Spark v1.6.1 or v1.6.2. Spark and the META-pipe executable require Scala v2.10.6. In addition, the META-pipe execution environment requires the Java v1.8 OpenJDK. Here we describe how META-pipe is set up on the cPouta cloud. The setup on other clouds may differ as described below. Additional details, including instructions for using the cluster setup tool are in the META-pipe cloud setup design document.

### META-pipe virtual machines and storage

A META-pipe execution environment has three types of virtual machines (VMs):

- *Bastion VM*: acts as the gateway machine used for cluster management and gateway to an initiated cluster.
- *Cluster Master VM*: acts as Spark Master, NFS Server, and it runs the main META-pipe executable.
- *Cluster Worker VMs*: act as Spark Workers, NFS Clients, and the runners of parallelized tasks of META-pipe jobs.

In addition, META-pipe requires the use of *NFS-shared storage* used by the worker VMs to read and write temporary computation data including intermediate result files. The master VM contains a NFS-server that serves the access to the storage. The worker VMs are NFS-clients that have full read-write access to the shared storage. The NFS server also has the Spark and Scala installations. The NFS storage can be either a Master VM internal volume, or a virtual volume attached to the Master. We typically use the latter, since it makes deployment easier and it allows META-pipe volume caching (as described below). We have, however, not compared the performance of these two approaches.

### META-pipe executable and dependencies

The META-pipe executable is downloaded from our artifacts server. The executable is a (42 Megabyte) jar file that contains the Spark job, and it is submitted as a Spark job from the Master VM. The executable jar does not contain 3 ^rd^ party tools used by the pipeline, tool libraries, and the reference databases used by the tools. These dependencies must be downloaded from our artifacts web server. The dependencies are 44 Gigabytes, of which most of the space is used for the reference databases. Each worker VM must have access to the META-pipe dependencies, so they are typically stored locally on each cluster VM. If there are not enough volume resources, NFS storage is used.

### Tests

After setting up the Spark, a simple parallelized script should be used to test that Spark and META-pipe is set up correctly. We first test Spark by submitting a parallelized version of prime number counter, wait for all Workers to be done, and then ensure that there were no error messages and that the result is correct.

To validate the correctness of META-pipe installation, we use the built-in validation procedure in the META-pipe executable. This procedure will check the state of all tools required and their dependencies that are required for META-pipe execution. It will also check that the tools do not return errors.

### META-pipe job execution

After initialization, the submitted executable will listen for, and run, new jobs until the spark-submit is stopped. The jobs are submitted to the central META-pipe job server that checks the tag in the job and submits it to a specific META-pipe executable. The executable downloads the input data from the META-pipe storage server to a Spark RDD data structure and launches the META-pipe job using the spark-submit command. When the job is completed, the executor uploads the output datasets to the META-pipe storage server, which are then accessible by the user on META-pipe portal. After the execution of each tool in the pipeline, the intermediate output datasets are also uploaded to the storage server, so that if the job fails, it can be restarted from the last successful pipeline step.

### cPouta Open Stack setup

To set up the META-pipe execution manager on cPouta we created an execution manager setup tool that setups the virtual machines, storage, Spark, and META-pipe as described above. It is implemented as a command line tool written in Java, with some parts implemented in Bash, Ansible and Python. The tool’s requirements, usage information, as well as more detailed technical information is in our design document.

We have optimized cluster provision by caching virtual volumes with the META-pipe execution manager and dependencies. To avoid downloading, unpacking and preparing META-pipe files for each new cluster instance, we store these in a virtual volume the first time a cluster is set up. This volume is used as the storage of prepared META-pipe files (a cache), which is used to create volumes for cluster VMs in later cluster provision. This reduces the time to create a cluster from 30 minutes to 10 minutes. In comparison, the execution time for a typical META-pipe job is several hours.

### CESNET-MetaCloud OpenNebula setup

To set up the META-pipe execution manager on the CESNET-MetaCloud cloud we adapted the cPouta tool to create a tool that uses the Open Cloud Computing Interface ( OCCI). It is therefore compatible with all EGI Federated Clouds, since they all support OCCI. The tool is a rOCCI Client implemented in Python and ansible that uses X509 VOMS certificates. It implements a Terraform OCCI plugin. Additional details are in https://github.com/cduongt/mmg-cluster-setup-CESNET.

The manager of the server must provide a contextualization file and Terraform configuration file that define the technical features of the virtual cluster. When the launching command is issued the tool first builds the virtual cluster to the given endpoint and then automatically installs the software components and reference datasets to the new virtual cluster as described above.

The end users submit analysis tasks from the META-pipe web app to the META-pipe backend running in EGI Federated cloud. The end users do not need certificates, Virtual Organization membership or the tools required to launch the META-pipe backend. Instead, the end users just authenticate to the META-pipe web interface using ELIXIR AAI.

### Elastic MapReduce on Amazon Web Services

To set up the META-pipe execution manager on Amazon Web Services (AWS), we use the AWS Elastic MapReduce (EMR) console and a custom cluster boot-time script. We use AWS’s EMR managed framework since it natively supports Spark. The cluster setup is therefore simpler than on OpenStack and OpenNebula, but it is not as configurable. For instance, EMR clusters always use the YARN resource allocator and cannot be configured to use Spark’s “standalone” mode instead, which we use on the other platforms. This has not been a big problem in practice, but it constitutes an uncontrollable variable when optimizing the execution for various cloud platforms. A detailed description of the setup is in https://gitlab.com/uit-sfb/metapipe-on-aws. We plan to implement a setup tool like the OpenStack and EGI Federated Cloud tools described above. For automation of EMR cluster setup, AWS offers a comprehensive application programming interface (API) and command line interface (CLI), and CloudFormation.

For META-pipe, we make AWS EMR clusters on demand. Our clusters use spot instances with VM flavors that provide the best cost-performance for META-pipe (currently we use c4.4xlarge). An EMR cluster boots up with a compatible version of Spark provisioned by Amazon. Our automatically started boot-time script then provisions META-pipe tools and dependencies from an S3 bucket. The whole cluster creation process takes about 10 minutes. Similar to other execution environments, the cluster is idle until we start the META-pipe execution manager on the cluster’s master node. The execution manager continually fetches jobs submitted using the META-pipe web app and submits them to Spark on the cluster. We must terminate the cluster ourselves, either by a script through the AWS API or manually in the EMR console.

## Use cases

Here we describe a use case where the computationally demanding parts of META-pipe are setup to execute on a cloud resource provided for a new user community. The new user group first applies for computational resources from its partner clouds, and then utilizes these resources easily through the interface provided by the META-pipe web application. The tools described here makes it easy for a resource provider to administer the META-pipe job executions, and the use of standardized technologies and protocols ensure compatibility and portability of the META-pipe analysis backend across clouds. Below we describe the execution of analysis jobs from the point of view from META-pipe end users, compute resource providers, and META-pipe service providers. We have also described these use cases in an ELIXIR webinar (November 2016) about the ELIXIR compute platform.

### End user

The new user community, represented for example by their national ELIXIR node, have applied and received compute resources from an academic cloud provider. One of the academic users has a marine metagenomics dataset they want to analyze:

1. The user logs into the META-pipe web application using their home institution credentials. The login page is the single sign on provided by ELIXIR AAI. The datasets are typically up to a few GB in size and they can quickly be uploaded using the browser.
2. The user uploads their dataset to be analyzed, and possibly changes some of the analysis parameters. This is done in the META-pipe web app.
3. The user tags the cloud to use for the analysis and submits the job for analysis using the web app GUI.
4. Once computations finish, the data is returned to the portal, and the user can download the enriched results for further analysis or visualization using separate tools such as Krona ^2^, Artemis ^3^, or METAREP ^4^.

### Resource provider

The resource provider must setup the META-pipe execution manager that executes the Spark job that implements the analysis. In addition, the resource provider must test and maintain the execution manager. The execution manager setup tools described in the previous section simplifies this task.

The first time the META-pipe backend is set up on a cloud environment, the resource administrator needs to edit a configuration file that defines the virtual cluster to be created. After that the administrator runs the following commands in the tool:

1. *create-env:* to set up the environment and META-pipe files caching volume that will be used in create-cluster.
2. *create-cluster*: provision cluster resources, install and configure the execution manager (Spark and NFS), install META-pipe tools, dependencies, and reference databases on the provisioned cluster VMs, and test the setup.

To set up a cluster as second time, only step 2 is run. It will use cached META-pipe volumes created previously.

Then to accept META-pipe jobs from the job server:

3. *sw-launch*: start Spark and the server that listens for new META-pipe jobs to execute.

To stop accepting new jobs:

4. *sw-kill*: stop all META-pipe related processes on all cluster VMs.

To free the allocated resources:

5. *remove-cluster*: remove the cluster and keep the environment for future use.
6. *remove-env*: remove the environment, including cached volumes.

Step 6 is only done when the resource is not intended to be used for META-pipe jobs anymore. Steps 2–5 may be automated.

### Service provider

The META-pipe team providing the service do not need to make any changes to the central services since the new execution manager is authorized using the META-pipe authorization server, and since the end-user specifies the tag for the new execution manager.

### Metagenomics course

In April 2017 we used META-pipe in a metagenomics course organized by the Finnish ELIXIR node ( https://www.csc.fi/web/training/-/metagenomics also described in the report “ EGI-Engage D6.15 Demonstrator for ELIXIR workflows implemented in the EGI Federated cloud”). We setup two META-pipe execution environments. The main execution environment was running in the cPouta cloud environment at CSC, and a backup execution environment that was running in EGI Federated Cloud (CESNET-MetaCloud). These META-pipe backends were set up by the course organizers and so the students did not need to do any technical preparations to use the cloud services. Instead, they only needed to define one extra parameter in the web interface to guide their analysis tasks to a specific external META-pipe backend. 42 students participated to the metagenomics course and successfully used these temporary execution environments through the META-pipe web interface without any interference.

## Discussion

### Related work

Bioinformatics pipelines can be specified for portable execution in either a popular bioinformatics pipeline (workflow) manager, such as Galaxy ^5^ or Chipster ^6^, or in a standardized language, such as the Common Workflow Language ^7^, that is supported by many pipeline managers including Galaxy and Toil ^8^. META-pipe is implemented in Apache Spark. Spark is widely used for big data processing, and it is supported natively in Amazon Web Services, Microsoft Azure, and Google Cloud Platform.

Another approach for portable bioinformatics tool execution is to package the tools as containers. Repositories such as BioContainers provides many tools. Several pipeline managers support containers, including Nextflow ^9^, Toil, Pachyderm, and Luigi ^10^. META-pipe does not use containers, since our artifacts server and the ansible scripts used by the setup tools take care of META-pipe dependencies. In addition, even when using containers, there is a need to set up container orchestration for parallel execution on distributed resources. Systems such as Kubernetes and Docker Swarm can be used to orchestrate containers. The META-pipe execution manager use Spark for orchestration.

The EBI Cloud Portal enables execution of pipelines on cloud resources. Users can sign on using ELIXIR AAI, add their applications, pipelines as virtual machine images, and configure cloud compute and storage resources. We attempt to hide these details to the end users. Commercial solutions include platform such as Illumina BaseSpace, where developers can provide apps for analysis on AWS of data uploaded to BaseSpace. Currently, most of the provided apps are single tools instead of complete pipelines, such as META-pipe.

### Limitations

An important limitation of our approach is that we do not handle resource allocation for end users. Instead users must contact a service provider in their country (or be added to an EGI based virtual organization (VO)) to allocate resources and setup an execution environment. This is not something all users know how to do, and it is unnecessarily complicated for small projects. There are four possible solutions. First, for small projects an ELIXIR node may provide the computation resources for all users. Second, an ELIXIR node may provide computation resources for all their users. Third, federated cloud resources can be used through EGI Federated Cloud or the ELIXIR federated cloud testbed. Long term usage of federated approach requires that access guarantees (service level agreements, operational level agreements) are arranged. Fourth, the users can allocate and pay for resources from a commercial cloud provider. Such pay-by-use is especially easy for industry users.

We do not provide a service for predicting resource usage for META-pipe jobs. However, we believe that we can make good estimates based on the input file size. We are also currently evaluating and optimizing META-pipe job execution. An important part of such optimization is to choose the most cost efficient virtual machine flavors and storage solutions on a cloud.

## Conclusion

We have described our approach for setting up and executing the functional analysis of META-pipe on academic and commercial clouds. To make our analysis service easy to use and to maintain, we use a distributed architecture where we combine central servers with multiple distributed backends that execute the computationally intensive jobs. A key issue in ELIXIR is to obtain computing resources for META-pipe end users. It is still to be decided how resources will be obtained, managed and allocated to the individual end users. For all solutions, an up-to date and highly automatized tool for launching a META-pipe execution environment is needed. We will continue improving the META-pipe backend and to add support for additional resource providers.

## Data and software availability

No data is needed to use the cloud setup tools.

The code for the three setup tools are open source at:

- CSC cPouta OpenStack cloud: https://gitlab.com/uit-sfb/METApipe-cPouta-cloud-setup.
- CESNET-MetaCloud OpenNebula: https://github.com/cduongt/mmg-cluster-setup-CESNET.
- Amazon Web Services EMR: https://gitlab.com/uit-sfb/metapipe-on-aws.

Archived code at time of publication for all: http://doi.org/10.5281/zenodo.1053807 ^11^. All use the MIT license. The above repositories include user guides.
