# Supplementary material for: META-pipe cloud setup and execution
Source: F1000Res. 2019 May 2;6:ELIXIR-2060. Originally published 2017 Nov 29. [Version 3] doi: 10.12688/f1000research.13204.3 (PMC6480938; doi:10.12688/f1000research.13204.3)
Supplement: Peer review history

# Peer review: Reviewer response for version 2

**DOI:** 10.5256/f1000research.14965.r46505

**Published:** 2019-04-23

**Authors:** Chunlei Wu

**Affiliations:** 1 Department of Molecular and Experimental Medicine (MEM), Scripps Research Institute, La Jolla, CA, USA

This manuscript described META-pipe as a cloud-based workflow for marine metagenomic analysis. The computation-intensive analysis is parallelized via a distributed Spark-compatible infrastructure. The unique feature of META-pipe is its support of heterogeneous cloud environment for job execution, including those available from academic institutes and commercially available AWS.

 This is the 2nd version of the manuscript. The authors have addressed all remarks raised by the previous reviewers.

 A few minor remarks are listed below:

 Use cases->End user section:

- Cannot evaluate the application without a valid ELIXIR AAI login credential. Should either provide a demo account or a recorded screencast for the review purpose.

Discussion->Limitations section:

- "(or be added to an EGI based VO)": What's "VO"?
- "requires that access guarantees (SLAs, OLAs)": the first appearance of acronyms (SLAs, OLAs) should be spelled out and/or include links.

I have read this submission. I believe that I have an appropriate level of expertise to confirm that it is of an acceptable scientific standard.

# Response

**Published:** 2019-04-26

**Authors:** Lars Ailo Bongo

**Affiliations:** University of Tromso, Norway

Thank you for your suggestion to improve the readability of our paper. We have now spelled out the acronyms. Also note that in the "Use cases" section we provide a link (https://elixir-europe.org/documents/elixir-webinar-elixir-compute-platform-roapmap-november-2016) to a seminar where we show the end user interfaces.

# Peer review: Reviewer response for version 1

**DOI:** 10.5256/f1000research.14327.r29076

**Published:** 2017-12-22

**Authors:** Kang Ning

**Affiliations:** 1 Department of Bioinformatics and Systems Biology, Huazhong University of Science and Technology, Wuhan, China

This article describes the setup of a multi-cloud architecture that can support the execution of META-pipe jobs for marine metagenomic data analysis. And META-pipe provides assembly of high-throughput sequence data, functional annotation of predicted genes, and taxonomic profiling.

**R** **emarks:**

1. The introduction of META-pipe backend architecture and deployment is detailed and clear
2. It achieves the goal which is to provide a powerful analysis service that is easy to use and to maintain since META-pipe has web app and GUI that the users just set parameters and then they can make analysis of data.
3. In the chapter concerning resource provider (page 6), “The tools described in the previous section simplifies this task“ needs more details like names of the tools since there are some tools referred in the previous section. It would be easy to understand.
4. There is less example to indicate tasks in META-pipe can be easy and fast. It would be better to give some data like running time and volume to state. Giving other projects differed from this as ‘ control group ’and setting characters to make comparisons will be more persuasive.
5. There are some spelling mistakes like ‘manger’.

I have read this submission. I believe that I have an appropriate level of expertise to confirm that it is of an acceptable scientific standard, however I have significant reservations, as outlined above.

# Response

**Published:** 2018-01-16

**Authors:** Lars Ailo Bongo

**Affiliations:** University of Tromso, Norway

Thank you for your remarks. We have specified that the tools used by the resource provider are the execution manager setup tools.

 Performance and scalability evaluation of META-pipe on different execution environments is important, but outside the scope of this paper. Here we describe the work done before and after executing pipeline jobs. This approach can be used for other bioinformatics pipelines (implemented as Spark jobs). A performance evaluation would require a detailed explanation of the META-pipe tools. To make this point clearer we have added a line in “cPouta Open Stack setup” about META-pipe job execution time being several hours (compared to 10-30 minutes of setup time).

# Peer review: Reviewer response for version 1

**DOI:** 10.5256/f1000research.14327.r28995

**Published:** 2017-12-15

**Authors:** Olivier Collin

**Affiliations:** 1 Univ Rennes, Inria, CNRS, IRISA F-35000, Rennes, France

The article describes the setup of a multi-cloud architecture that can support the execution of META-pipe jobs for marine metagenomics data analysis.

 It is intended for a technically inclined audience and focuses on the different configuration steps required for the setup of a complex computing architecture involving several clouds in several countries.

 The purpose is to be able to open the META-pipe service to a large number of ELIXIR users.  Several cloud middleware have been configured (Openstack, OpenNebula, Amazon).

 The description of the infrastructure is clear and additional links to github repositories or online documents allow the reader to fetch extra technical information.

 Remarks :

 In the introduction, the authors indicate that the design goal is to make execution mangers (sic) portable and that the setup and maintenance of execution mangers (sic) is easy. The authors state that these goals are achieved because all state is maintained on central servers. This section is not completely clear and should be rewritten to highlight the advantages of the central servers architecture. Emphasizing the fact that the META-pipe environment will be pushed from a central server to the execution clouds could clarify things.

 In the chapter concerning the META-pipe executables and dependencies (page 4), it could be interesting to better describe these dependencies. These dependencies are downloaded from the artifact server but no technical information is given about this server (for example : location, size of the downloads). Are the reference databases provided by the artifact server ?

 The infrastructure is articulated around a central server hosting the configuration of META-pipe. This creates a single point of failure. It could be interesting to describe the actions that are taken in order to have a more resilient system.

 In the META-pipe job execution paragraph, the input data is downloaded to a data structure (page 5, col 1, line 18). What is this data structure ?

  Typos:

 Page 3 / Col 2 / Line 4, 5, 11 : manager/manger

 Page 3 / Col 2 / Line 27 : executables instead of executable

 Page 3 / Col 2 / Line 29 : is set up

 Page 5 / Col 1 / Line 3 : is set up

 Page 5 / Col 1 / Line 27 : to set up

 Page 5 / Col 2 / Line 42 : are set up

 Page 5 / Col 2 / Line 45 : utilizes

 Page 6 / Col 1 / Line 13 : its dataset

 Page 6 / Col 1 / Line 23 : set up

 Page 6 / Col 1 / Line 28 : set up

 Page 6 / Col 1 / Line 38 : To set up a cluster for a second time

 Page 6 / Col 2 / Line 19  : We set up

 Page 6 / Col 2 / Line 24 : did not need to :  Missing word

 Page 6 / Col 2 / Line : several pipeline mangers : managers ?

 Page 7 / Col 1 / Line 19 : ELIXIR

I have read this submission. I believe that I have an appropriate level of expertise to confirm that it is of an acceptable scientific standard.

# Response

**Published:** 2018-01-16

**Authors:** Lars Ailo Bongo

**Affiliations:** University of Tromso, Norway

Thank you for your remarks. We have made the following changes in our revised version:

1. We improved the description of the centralized server and the execution environments in the introduction.
2. We added additional details about the dependencies and the artifacts server in “META-pipe executable and dependencies”
3. We now specify that the data is downloaded into a Spark RDD.
4. We have fixed the typos.

We agree that centralized servers create a single-point-of-failure, but at the same time it simplifies the implementation of the backend. We are not a stage at the project yet where we believe the improved availability justifies prioritizing this issue.
